# Facultatively Anaerobic Staphylococci Enable Anaerobic *Cutibacterium* Species to Grow and Form Biofilms Under Aerobic Conditions

**DOI:** 10.3390/microorganisms12122601

**Published:** 2024-12-16

**Authors:** Jeffrey B. Kaplan, Michael Assa, Noor Mruwat, Miloslav Sailer, Suresh Regmi, Khalaf Kridin

**Affiliations:** 1 Laboratory for Skin Research, Institute for Medical Research, Galilee Medical Center, Nahariya 2210001, Israel; noorm2@gmc.gov.il (N.M.); dr_kridin@hotmail.com (K.K.); 2 The Azrieli Faculty of Medicine, Bar-Ilan University, Safed 1311502, Israel; michael.assa@biu.ac.il; 3 Kane Biotech Inc., Winnipeg, MB R3T 6G2, Canada; msailer@kanebiotech.com (M.S.); sregmi@kanebiotech.com (S.R.)

**Keywords:** acne vulgaris, *Cutibacterium acnes*, dispersin B, DNase I, dual-species biofilm, extracellular DNA, PNAG, skin microbiome

## Abstract

Facultatively anaerobic *Staphylococcus* spp. and anaerobic *Cutibacterium* spp. are among the most prominent bacteria on human skin. Although skin microbes generally grow as multispecies biofilms, few studies have investigated the interaction between staphylococci and *Cutibacterium* spp. in dual-species biofilms. Here, we measured the mono- and dual-species biofilm formation of four staphylococcal species (*S. epidermidis*, *S. hominis*, *S. capitis*, and *S. aureus*) and two *Cutibacterium* spp. (*C. acnes* and *C. avidum*) cultured in vitro under both aerobic and anaerobic conditions. The biofilms were quantitated by rinsing them to remove planktonic cells, detaching the biofilm bacteria via sonication, and enumerating the cells by dilution plating. When cultured alone, staphylococci formed biofilms under both aerobic and anaerobic conditions, whereas *Cutibacterium* spp. formed biofilms only under anaerobic conditions. In co-culture, staphylococcal biofilm formation was unaffected by the presence of *Cutibacterium* spp., regardless of oxygen availability. However, *Cutibacterium* spp. biofilm formation was significantly enhanced in the presence of staphylococci, enabling robust growth under both anaerobic and aerobic conditions. Fluorescence confocal microscopy of the aerobic dual-species biofilms suggested that staphylococci create anaerobic niches at the base of the biofilm where *C. acnes* can grow. These findings demonstrate that staphylococci facilitate the colonization of *Cutibacterium* spp. in oxygen-rich environments, potentially explaining their presence in high numbers on the oxygen-exposed stratum corneum.

## 1. Introduction

The Gram-positive bacteria *Cutibacterium acnes* and *Staphylococcus epidermidis* are among the most abundant members of the human skin microbiome [1,2]. Although both species are considered beneficial commensals because they help maintain homeostasis of the skin microbiota and control pathogens such as *S. aureus* via colonization resistance [3,4,5], both species can also act as opportunistic pathogens. *C. acnes* causes invasive infections of the skin, soft tissue, and cardiovascular system [6], while *S. epidermidis* is associated with bacteremia in preterm infants and atopic dermatitis [5]. Both species also cohabitate in acne vulgaris lesions [7,8] and cause serious infections of implanted medical devices [6,9]. *C. acnes* and *S. epidermidis* are frequently co-isolated from clinical skin samples [10] and medical device infections [11].

Because of their shared abundance in the skin microbiome community, *C. acnes* and *S. epidermidis* likely evolved a close relationship based on competition and mutualism. For example, previous studies showed that *C. acnes* competes with *S. epidermidis* in hair follicles through the production of the antimicrobial peptide cutimycin [12]. *C. acnes* may also inhibit *S. epidermidis* biofilm formation and sensitize *S. epidermidis* to antibiotic killing through the production of short-chain fatty acids [13]. *S. epidermidis*, however, can counteract this competition through the production of antimicrobial peptides and the fermentation of glycerol into short-chain fatty acids that suppress *C. acnes* growth [14].

In addition to interspecies competition, *C. acnes* and *S. epidermidis* likely evolved the ability to mutually coexist in polymicrobial biofilms, which are the primary mode of growth for bacteria on human skin [15]. Biofilms are defined as densely packed layers of bacterial cells that grow attached to a tissue or surface [16]. Bacteria in a biofilm are encased in a self-synthesized, extracellular polymeric matrix that holds the cells together in a mass, attaches them to the underlying surface, and protects them from killing by antimicrobial agents and host immunity. In vivo, *C. acnes* biofilms have been observed in acne lesions and on implanted medical devices [17,18], and *S. epidermidis* biofilms have been observed on skin, in sweat glands, and on implanted medical devices [19,20]. Both *C. acnes* and *S. epidermidis* form biofilms in vitro [6,9]. Biofilms play a role in many chronic infections which are often difficult to treat because of the protective nature of biofilms [16].

Among the few studies that have investigated *C. acnes*/*S. epidermidis* dual-species biofilms, the biofilms were cultured under anaerobic conditions [21,22,23]. This is because *C. acnes* is an aerotolerant anaerobe whose growth is inhibited by oxygen. No studies investigating *C. acnes*/*S. epidermidis* dual-species biofilms under aerobic conditions have been reported. Aerobiosis more closely mimics the environment on the stratum corneum, which is directly exposed to atmospheric oxygen, and in hair follicles and sebaceous glands, where oxygen levels are often only moderately hypoxic due to diffusion of oxygen from the atmosphere and tissues [24].

In the present study, we assessed the ability of *S. epidermidis* and *C. acnes*, either individually or jointly, to form biofilms in vitro. Since *S. epidermidis* is a facultative anaerobe and *C. acnes* is an aerotolerant anaerobe, we incubated culture vessels under both aerobic and anaerobic conditions to determine the role of atmosphere in biofilm formation. Our findings suggest that *S. epidermidis* significantly enhances growth and biofilm formation by *C. acnes* under both anaerobic and aerobic conditions. These findings may be clinically relevant to the interaction between staphylococci and *Cutibacterium* spp. on human skin.

## 2. Materials and Methods

### 2.1. Bacterial Strains

The bacterial strains used in this study are listed in Table 1. *C. acnes* strain HL086PA1 was obtained from BEI Resources (Manassas, VA, USA) as part of the NIAID NIH Human Microbiome Project. *C. acnes* strains 2, 7, and 14, and *C. avidum* strain 42, were provided by Ronen Hazan and Amit Rimon (Hebrew University of Jerusalem, Jerusalem, Israel). *S. epidermidis* strain 5 was obtained from Irina Sadovskaya (Université du Littoral-Côte d’Opale, Boulogne-sur-mer, France). *S. epidermidis* strain 1457 and green fluorescent protein-expressing strain 1457/pCM29-GFP were provided by Holger Rohde (Universitätsklinikum Hamburg-Eppendorf, Hamburg, Germany). *S. aureus* strain JE2 was kindly provided by Alex Horswill (University of Colorado, Boulder, CO, USA). Staphylococcal laboratory strains JK4, JK5, JK6, JK7, JK9, and JK10 were identified at the species level using MALDI-TOF mass spectrometry.

### 2.2. Media and Growth Conditions

The media used were tryptic soy agar (TSA) and tryptic soy broth (TSB), both purchased in powder form from Becton, Dickinson and Company. All bacteria were cultured at 37 °C. The reference stock cultures were maintained at −80 °C in 20% glycerol/80% TSB. The working stock cultures were restreaked weekly on TSA. Staphylococcal working stocks were incubated for 1 d in air. *Cutibacterium* working stocks were incubated for 3 d under anaerobic conditions generated with a BD GasPak EZ Anaerobe sachet system.

### 2.3. Test Tube and Microtiter Plate Biofilm Assays

A loopful of cells from a fresh agar plate was transferred to a 1.5 mL microcentrifuge tube containing 200 µL of sterile TSB. The cells were mixed by vortex agitation and then diluted to 10^6^–10^7^ CFU/mL in filter-sterilized TSB (1:1000 dilution). Dual-species biofilms were inoculated at a *C. acnes*/*S. epidermidis* ratio of 1–8:1. Inocula were aliquoted into 13 × 100 mm glass tubes (1 mL/tube), 15 mL conical-bottom polypropylene tubes (1 mL/tube), 96-well round-bottom non-tissue-culture-treated polystyrene microtiter plates (0.2 mL/well), or 24-well flat-bottom tissue-culture-treated polystyrene microtiter plates (1 mL/well). Cultures were incubated statically for 24–96 h in air or under anaerobic conditions as described above. In some experiments, polypropylene tubes were incubated under dynamic conditions by clamping them in a nearly horizontal position in a roller apparatus set to 40 rpm, which inhibited the settling of cells at the bottom of the tube and biofilm formation.

### 2.4. NHEK Cell Culture

Normal human epidermal keratinocytes (NHEKs) were purchased from ATCC (# PCS-200-011). The cells were cultured in 24-well tissue-culture-treated microtiter plates. Then, the cells were seeded at a density of 5 × 10^4^ cells per well in 500 µL of dermal cell basal medium (ATCC # PCS-200-030) supplemented with Keratinocyte Growth Kit components (ATCC # PCS-200-040) and antibiotics, as per the supplier’s recommendations. The cells were incubated at 37 °C in a humidified atmosphere with 5% CO_2_. The medium was replaced every 48 h. Monolayers were allowed to reach approximately 100% confluence, which typically occurred in 5–6 d. Prior to bacterial inoculation, the cells were washed twice with sterile phosphate-buffered saline (PBS) to remove residual medium, antibiotics, and non-adherent cells.

### 2.5. Enumeration of Biofilm Bacteria

For the glass tubes, the biofilms were rinsed three times with 3 mL of PBS, and the tubes were filled with 1 mL of PBS. The biofilm bacteria were detached from the walls of the tubes by sonication for 30 s at 50% power/50% duty cycle using an IKA Labortechnik sonicator equipped with a microtip probe. Control experiments showed that this sonication treatment did not affect the viability of the *C. acnes*, *S. epidermidis*, or *S. aureus* cells. The sonicates were serially diluted in PBS. To selectively enumerate staphylococcal CFUs, dilutions were plated on TSA and incubated in air. To selectively enumerate *Cutibacterium* CFUs, dilutions were plated on TSA supplemented with 20 µg/mL erythromycin and incubated anaerobically. All *Cutibacterium* strains used in this study were erythromycin-resistant, and all staphylococcal strains were erythromycin-susceptible (Table 1). For the polypropylene tubes, the tubes were mixed for 10 s at a high speed in a Vortex Genie 2 mixer and then subjected to sonication for 15 s at 50% power in a Qsonica CL188 sonicator equipped with a microtip probe. Control experiments showed that the 10 s vortex agitation step removed nearly all staphylococcal biofilm cells and approximately 10% of the *Cutibacterium* biofilm cells from the polypropylene tubes, and that the 15 s sonication step efficiently detached the remaining *C. acnes* biofilm cells without affecting the staphylococcal or *Cutibacterium* cell viability The sonicates were diluted and plated on selective agar for CFU enumeration, as described above. For the 96-well microtiter plates, the contents of each well were mixed vigorously by pipetting up and down 20 times in combination with stirring and scraping the bottoms of the wells using the pipette tips. The contents of each well were then diluted and plated on selective agar for CFU enumeration as described above. For the 24-well microtiter plates, the biofilms were scraped from the bottom of the well with a cell scraper and then mixed by vortex agitation, diluted, and plated on selective agar for CFU enumeration, as described above.

### 2.6. Treatment of Biofilms with Enzymes

*S. epidermidis* strain 5/*C. acnes* strain HL086PA1 dual-species biofilms were cultured aerobically in glass tubes. After 72 h, the biofilms were rinsed with water to remove planktonic cells, and then treated with PBS supplemented with 80 µg/mL dispersin B (Kane Biotech, Winnipeg, MB, Canada) or 100 µg/mL DNase I (Sigma-Aldrich, St. Louis, MO, USA). Control biofilms were treated with PBS alone. After 15 min at 37 °C, the biofilms were rinsed vigorously with tap water to remove detached cells and then stained for 1 min with 1 mL of Gram’s crystal violet. The tubes were then rinsed with tap water to remove the unbound dye, and then air-dried and photographed.

### 2.7. Colony Biofilm Assay

Steam-sterilized 25 mm diameter, 0.45 µm pore-size nylon filters (#66607, Pall Corp., Port Washington, NY, USA) were transferred to the surface of a TSA plate. A volume of 20 µL of inoculum was pipetted in the center of each filter. The inocula were *C. acnes* strain 2 alone (ca. 10^5^ CFUs), or *C. acnes* strain 2 plus *S. epidermidis* strain JK4 (ca. 10^5^ CFUs each). The plates were incubated aerobically or anaerobically for 3 d. The filters were transferred to 50 mL conical-bottom polypropylene centrifuge tubes containing 5 mL of PBS and sonicated for 20 s at 50% power in a Qsonica CL188 sonicator equipped with a microtip probe. The sonicates were diluted and plated on selective agar for *C. acnes* CFU enumeration, as described above.

### 2.8. Fluorescence Confocal Microscopy

A total of 4 mL of inoculum containing *C. acnes* strain 2 (5 × 10^6^ CFU/mL), *S. epidermidis* strain 1457/pCM29-GFP (5 × 10^6^ CFU/mL), or a mixture of both strains (5 × 10^6^ CFU/mL of each species) was transferred to a 35 mm diameter glass-bottom Petri dish. *C. acnes* mono-species cultures were incubated anaerobically, and *S. epidermidis* mono-species and *C. acnes*/*S. epidermidis* dual-species cultures were incubated aerobically. After 3 d, the biofilms were rinsed vigorously with water and treated with 2 mL of FluoroMounter with DAPI aqueous mounting medium (Bio SB, Santa Barbara, CA, USA) overnight at 4 °C. The biofilms were viewed under a Lieca DMi8 inverted confocal microscope using a 63× oil objective. The GFP was visualized using a 488 nm laser. The DAPI was excited using an 405 nm laser, and its emission was recorded at 470 nm.

### 2.9. Statistics and Reproducibility of Results

Biofilm assays were performed in triplicate test tubes, in duplicate or triplicate microtiter plate wells, or on duplicate filters for the colony biofilm assay. All experiments were repeated at least three times on different days. All repetitions exhibited a similar range of values and similarly significant differences in the mean CFU/tube, CFU/well, and CFU/filter values among the experimental groups. The results from the representative experiments are shown in the figures. The significance of the differences between the means was calculated using a Student’s *t*-test. A *p* value < 0.01 was considered significant.

## 3. Results

### 3.1. S. epidermidis Enabled C. acnes to Form Biofilms in Glass Tubes Under Aerobic Conditions

**Figure 1:**
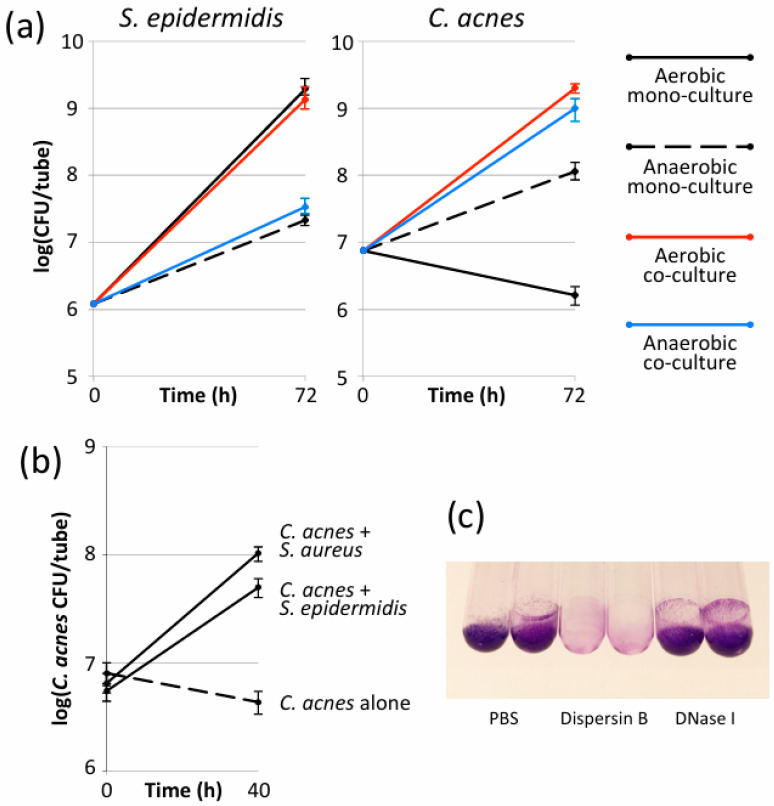
*Cutibacterium acnes* and *Staphylococcus* spp. mono- and dual-species biofilms grown in glass tubes under aerobic and anaerobic conditions. (**a**) Biofilm formation by *S. epidermidis* strain 5 (**left panel**) and *C. acnes* strain HL086PA1 (**right panel**) cultured alone (mono-culture—black solid and dashed lines) or together (co-culture—red and blue lines) under aerobic and anaerobic conditions. After 72 h, tubes were rinsed to remove planktonic cells, biofilms were detached by sonication, and species were individually enumerated by dilution plating on selective agar. Graphs show mean CFU/tube values for triplicate tubes. Error bars indicate sd. (**b**) Biofilm formation by *C. acnes* strain HL086PA1 cultured alone (dashed line) or co-cultured with *S. epidermidis* strain 5 or *S. aureus* strain JE2 (solid lines) for 40 h under aerobic conditions. Tubes were processed as in panel (**a**). Graph shows mean CFU/tube values for triplicate tubes. Error bars indicate sd. (**c**) Photographs of 72-h-old *S. epidermdis* strain 5/*C. acnes* strain HL086PA1 dual-species biofilms after treating them for 15 min with phosphate-buffered saline (PBS), PBS supplemented with dispersin B (80 µg/mL), or PBS supplemented with DNase I (100 µg/mL), and then staining them with crystal violet. Duplicate tubes for each treatment are shown.

Preliminary experiments were performed with *C. acnes* strain HL086PA1, an acne isolate, and *S. epidermidis* strain 5, an implant isolate (Table 1). When cultured individually in 13 × 100 mm glass tubes, *S. epidermidis* strain 5 formed biofilms under both anaerobic and aerobic conditions, whereas *C. acnes* strain HL086PA1 formed biofilms only under anaerobic conditions (Figure 1a). *S. epidermidis* strain 5 formed significantly more ßbiofilm under aerobic conditions than under anaerobic conditions (*p* < 0.01). When co-cultured, the presence of *C. acnes* strain HL086PA1 had no significant effect on biofilm formation by *S. epidermidis* strain 5 under either anaerobic or aerobic conditions (Figure 1a, left panel). However, the presence of *S. epidermidis* strain 5 significantly increased *C. acnes* strain HL086PA1 biofilm formation under anaerobic conditions (*p* < 0.01) and enabled *C. acnes* strain HL086PA1 to form robust biofilms under aerobic conditions (Figure 1a, right panel).

### 3.2. S. aureus Enabled C. acnes to Form Biofilms in Glass Tubes Under Aerobic Conditions

*C. acnes* also grew and formed biofilms in glass tubes under aerobic conditions when co-cultured with *S. aureus* strain JE2, a biofilm-forming MRSA strain (Figure 1b). This finding suggests that the biofilm enabling phenotype exhibited by *S. epidermidis* is a general phenomenon and not due to a specific interaction between *S. epidermidis* and *C. acnes*.

### 3.3. Aerobic S. epidermidis/C. acnes Biofilms Contained Poly-N-Acetylglucosamine Exopolysaccharide

Previous studies have shown that the exopolysaccharide poly-*N*-acetylglucosamine (PNAG) and extracellular double-stranded DNA (eDNA) function as adhesive components in both *S. epidermidis* and *C. acnes* biofilms [32,33,34]. To determine whether PNAG or eDNA contribute to biofilm cohesion in aerobic *S. epidermidis*/*C. acnes* dual-species biofilms, the biofilms were treated with the PNAG-degrading enzyme dispersin B [35], or with DNase I (Figure 1c). The treatment of dual-species biofilms with dispersin B, but not DNase I, resulted in a significant decrease in the crystal violet stainable biomass, suggesting that PNAG contributed to the cohesion of *S. epidermidis*/*C. acnes* dual-species biofilm under the conditions tested.

### 3.4. Multiple Staphylococcal Species Promoted Aerobic Biofilm Formation by C. acnes and C. avidum in Polypropylene Tubes

**Figure 2:**
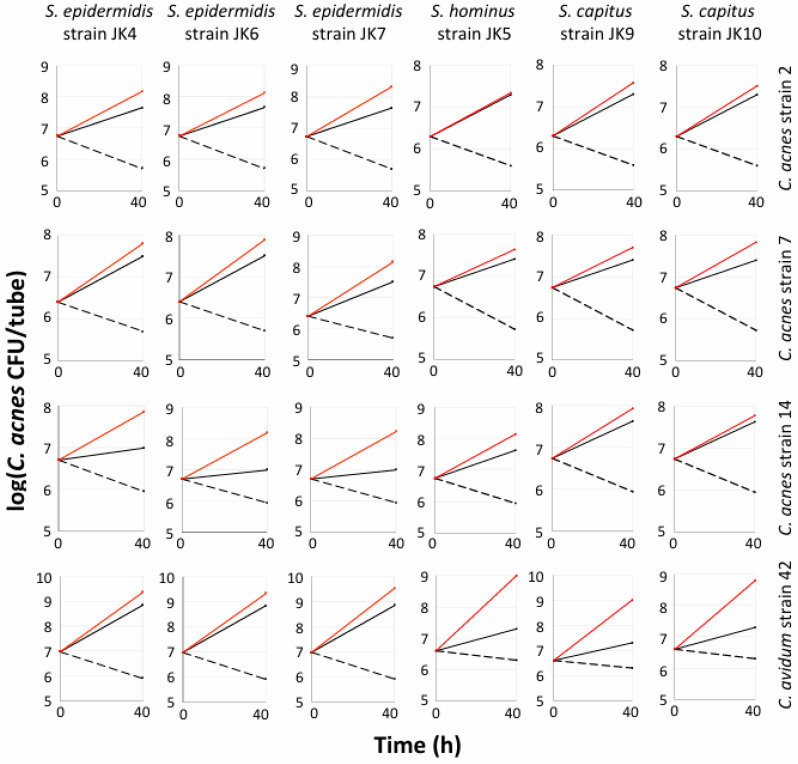
Growth of *Cutibacterium* spp. in 15 mL conical-bottom polypropylene tubes. Four *Cutibacterium* spp. (species and strain names indicated at right) were cultured alone anaerobically (solid black line), alone aerobically (dashed black line), or co-cultured aerobically with each staphylococcal species and strain indicated at top (red lines). Tubes were sonicated after 40 h of incubation, and sonicates were diluted and plated on agar plates selective for *C. acnes*. Graphs show mean *C. acnes* CFU/tube values for triplicate tubes. Error bars were omitted for clarity.

To investigate the generalizability of these results, we measured the biofilm formation of four *Cutibacterium* spp. strains (*C. acnes* strains 2, 7, and 14; *C. avidum* strain 42) when cultured alone under aerobic or anaerobic conditions, and when co-cultured with six different staphylococcal strains (*S. epidermidis* strains JK4, JK5, and JK7; *S. capitis* strains JK9 and JK10; *S. hominis* strain JK6) under aerobic conditions (Figure 2). These assays were carried out in 15 mL conical-bottom polypropylene tubes. As expected, when cultured alone, all four *Cutibacterium* spp. strains formed biofilms under anaerobic conditions but not under aerobic conditions. However, all four *Cutibacterium* spp. strains produced robust biofilms under aerobic conditions when co-cultured with any of the six staphylococcal strains. For all 24 combinations of strains, the amount of biofilm formed by *Cutibacterium* under aerobic conditions when co-cultured with *Staphylococcus* was equal to or greater than the amount formed in mono-culture under anaerobic conditions (Figure 2). This same phenotype was exhibited by *C. acnes* strain HL086PA1 and *S. epidermidis* strain 5 when co-cultured in glass tubes (Figure 1a). These results suggest that enabling *Cutibacterium* spp. to form biofilms under aerobic conditions is a common phenotype among staphylococci.

### 3.5. Aerobic Growth of C. acnes Depended on Biofilm Formation and Required Live S. epidermidis Cells

**Figure 3:**
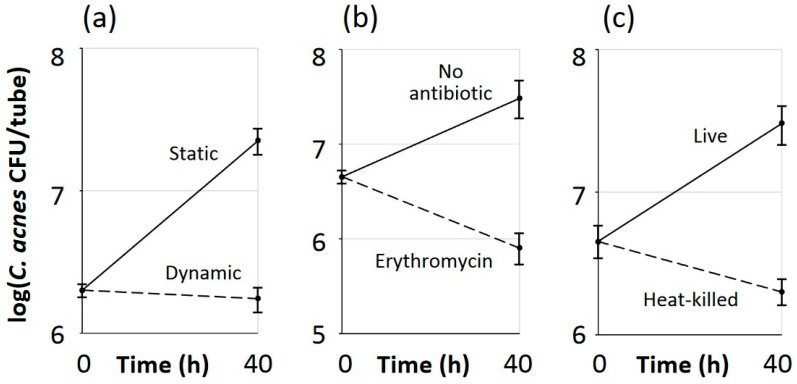
Growth of *C. acnes* strain 2 co-cultured with *S. epidermidis* strain JK4 in polypropylene tubes under aerobic conditions. After 40 h, tubes were sonicated, and sonicates were diluted and plated on selective agar for *C. acnes* CFU enumeration. (**a**) Tubes were incubated under static conditions or under dynamic conditions in a roller apparatus. (**b**). Media were supplemented with 20 µg/mL erythromycin or no antibiotic. (**c**) *S. epidermidis* was heat killed prior to inoculation. Graphs show mean CFU/tube values for triplicate tubes and error bars indicate sd.

Further experiments were performed utilizing *C. acnes* strain 2 (acne isolate) and *S. epidermidis* strain JK4 (healthy isolate). *C. acnes* strain 2 formed biofilms in air when co-cultured with *S. epidermidis* strain JK4 in polypropylene tubes only if the tubes were incubated under static conditions and not under dynamic conditions (in a roller apparatus), which inhibited biofilm formation (Figure 3a). Similarly, *C. acnes* strain 2 was unable to form biofilms under aerobic conditions when co-cultured with *S. epidermidis* strain JK4 if erythromycin was added to the culture medium to inhibit *S. epidermidis* growth (Figure 3b), or if the *S. epidermidis* inoculum was heat-killed prior to incubation (Figure 3c). These results suggest that the ability of *Staphylococcus* spp. to promote aerobic *Cutibacterium* biofilm formation requires live *S. epidermidis* cells and depends on de novo biofilm formation.

### 3.6. S. epidermidis Promoted Aerobic Growth of C. acnes in Polystyrene Microtiter Plate Wells but Not in Colony Biofilms

**Figure 4:**
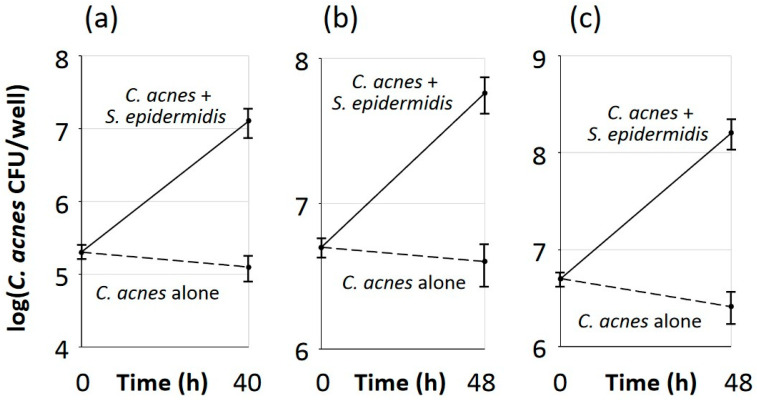
Growth of *C. acnes* strain 2 co-cultured with *S. epidermidis* strain JK4 in polystyrene microtiter plate wells. (**a**) *C. acnes* strain 2 was cultured alone (dashed line) or co-cultured with *S. epidermidis* strain JK4 (solid line) in 96-well, non-tissue-culture-treated, round-bottom microtiter plates under aerobic conditions. After 48 h, wells were scraped with a pipette tip, and contents of wells were diluted and plated on selective agar for *C. acnes* CFU enumeration. Graphs show mean CFU/well values for triplicate wells, and error bars indicate sd. (**b**) Same as (**a**), except bacteria were cultured in 24-well, tissue-culture-treated microtiter plate wells. (**c**) Same as (**b**), except surfaces of wells were seeded with NHEK cell monolayers.

*C. acnes* strain 2 exhibited robust growth under aerobic conditions when co-cultured with *S. epidermidis* strain JK4 in 96-well, non-tissue-culture-treated round-bottom polystyrene microtiter plates (Figure 4a), in 24-well, tissue-culture-treated flat-bottom microtiter plates (Figure 4b), and in 24-well microtiter plates that were seeded with NHEK cell monolayers (Figure 4c). The presence of *C. acnes* and *S. epidermidis* did not affect the morphology of NHEK cell monolayers as assessed by light microscopy. These findings demonstrate that *S. epidermidis* enabled the aerobic growth of *C. acnes* in multiple culture vessels and on multiple surfaces, including glass, polypropylene, polystyrene, and NHEK cell monolayers.

**Figure 5:**
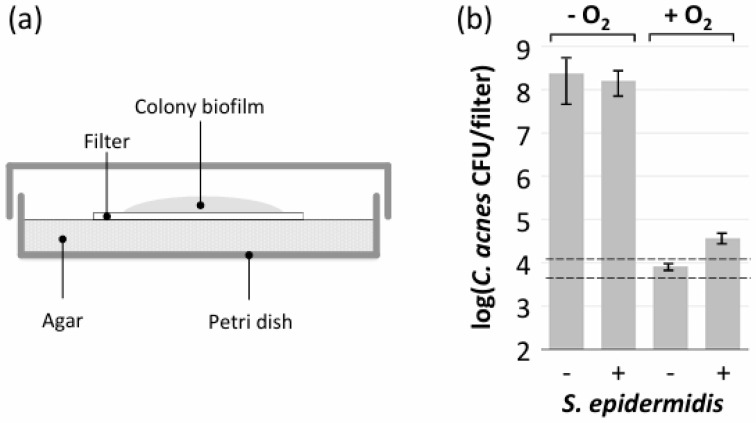
Colony biofilm assay. (**a**) Colony biofilms were cultured on a 25 mm diameter, 0.45 µm pore-size nylon filter placed on the surface of a tryptic soy agar plate. (**b**) *C. acnes* strain 2 was inoculated alone or in combination with *S. epidermidis* strain JK4 (indicated at bottom) onto filters. Plates were incubated anaerobically or aerobically (indicated at top) for 48 h. Filters were then transferred to 5 mL of saline and sonicated. Sonicates were diluted and plated on selective agar to enumerate *C. acnes* CFUs. Graph shows mean *C. acnes* CFU/filter values from a total of six filters from three independent experiments for each condition. Error bars indicate sd. Horizontal dashed lines indicate average starting *C. acnes* inoculum per filter (±1 sd).

Colony biofilms are biofilms that form on semipermeable membranes placed on the surface of an agar plate (Figure 5a). *C. acnes* strain 2 displayed robust growth in colony biofilms when cultured alone or co-cultured with *S. epidermidis* strain JK4 under anaerobic conditions (Figure 5b). However, under aerobic conditions, *C. acnes* strain 2 showed no growth in colony biofilms when cultured alone and only minimal growth when co-cultured with *S. epidermidis* strain JK4 (Figure 5b). Since colony biofilms are exposed to oxygen that diffuses through the agar and semipermeable membrane, this suggests that *S. epidermidis* requires an inert surface such as glass, polypropylene, or polystyrene to facilitate the aerobic growth of *C. acnes*.

### 3.7. C. acnes Outcompeted S. epidermidis After 3 Days of Growth Under Aerobic Conditions

**Figure 6:**
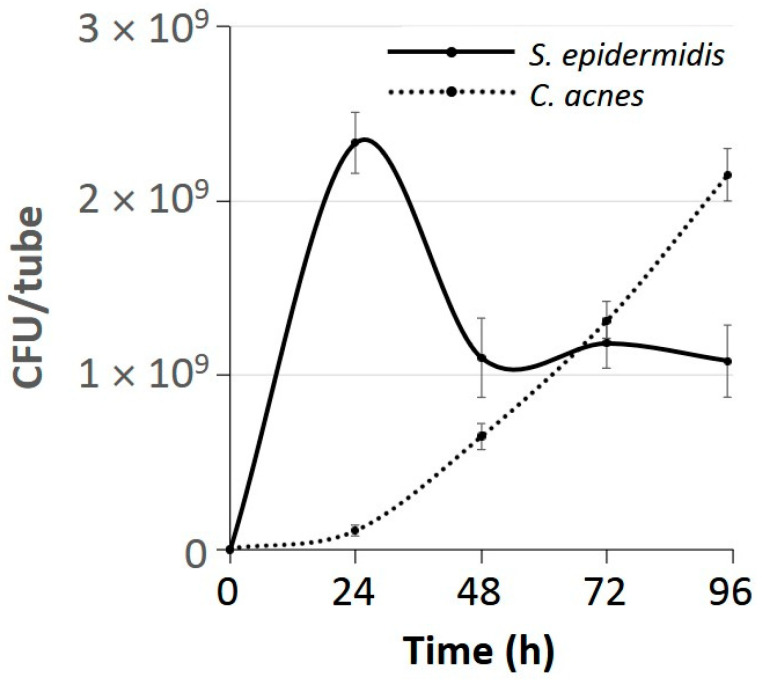
Growth of *C. acnes* strain 2 co-cultured with *S. epidermidis* strain JK4 in 15 mL polypropylene tubes under aerobic conditions. Values show mean CFU/tube of each species for triplicate tubes at each time point. Error bars indicate sd.

Time course studies revealed that after 3 d of aerobic growth, *C. acnes* strain 2 reproducibly achieved CFU/tube values that were equal to or greater than those achieved by *S. epidermidis* strain JK4 (Figure 6). The decrease in *S. epidermidis* CFU/tube values from 24–48 h occurred even in the absence of *C. acnes* strain 2 and was highly reproducible.

### 3.8. Aerobic C. acnes/S. epidermidis Dual-Species Biofilms Exhibited a Bilayer Structure

**Figure 7:**
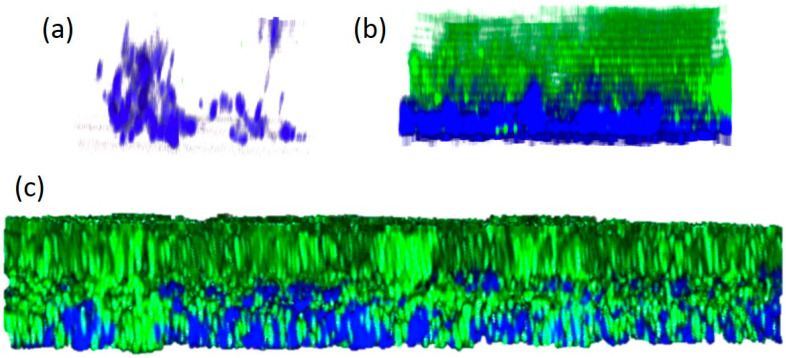
Cross-sections of *C. acnes* strain 2 and *S. epidermidis* strain 1457/pCM29-GFP biofilms grown on glass coverslips, stained with DAPI, and visualized by fluorescence confocal microscopy. (**a**) Anaerobic *C. acnes* mono-species biofilm. (**b**) Aerobic *S. epidermidis* mono-species biofilm. (**c**) Aerobic *C. acnes*/*S. epidermidis* dual-species biofilm. Biofilm thickness ≈ 20 µm. Green, GFP; blue, DAPI.

To investigate the structure of aerobic *C. acnes*/*S. epidermidis* dual-species biofilms, *C. acnes* strain 2 was co-cultured aerobically in glass-bottom Petri dishes with *S. epidermidis* strain 1457/pCM29-GFP, which expresses green fluorescent protein (GFP). As controls, *C. acnes* strain 2 was cultured alone under anaerobic conditions, and *S. epidermidis* strain 1457/pCM29-GFP was cultured alone under aerobic conditions. After 3 d, biofilms were stained with the DNA-binding dye DAPI, which fluoresces blue, and then analyzed by fluorescence confocal microscopy (Figure 7). As expected, anaerobic *C. acnes* strain 2 mono-species biofilms exhibited a signal for DAPI (blue) but not for GFP (Figure 7a). Aerobic *S. epidermidis* strain 1457/pCM29-GFP mono-species biofilms exhibited a bilayer structure, with green fluorescing aggregates comprising the entire top layer and blue fluorescing aggregates located exclusively in the bottom layer attached to the glass coverslip (Figure 7b). Aerobic *C. acnes* strain 2/*S. epidermidis* strain 1457/pCM29-GFP dual-species biofilms exhibited a bilayer structure like that exhibited by aerobic *S. epidermidis* strain 1457/pCM29-GFP mono-species biofilms (Figure 7c). In all biofilms, the predominant structures observed were independent green and blue fluorescing pillar- and tower-shaped aggregates in a 20 µm thick formation. Since eDNA is a component of *C. acnes* biofilms [32,36,37,38,39,40], these observations are consistent with the hypothesis that aerobic staphylococcal biofilms (green stain) create anaerobic niches at the base of the biofilm (blue stain) where *C. acnes* can grow protected from the bacteriostatic effects of oxygen.

## 4. Discussion

*Cutibacterium acnes* is one of the most prominent members of the human skin microbiome [41]. Ever since *C. acnes* was first isolated from acne lesions and grown in pure culture by the French physician Raymond Sabouraud in 1897 [42], it has always been cultured in the laboratory under anaerobic conditions, such as in a glove box or anaerobic jar. In this study, we found that anaerobic *C. acnes* and *C. avidum* strains can grow and form luxuriant biofilms in air when co-cultured with skin-associated staphylococci on glass, polypropylene, and polystyrene surface, and on NHEK cell monolayers. Remarkably, even though *C. acnes* is an anaerobe with a slow growth rate, it outcompeted fast-growing facultative *S. epidermidis* after three days of growth when both species were co-cultured in polypropylene tubes under aerobic conditions (Figure 5).

Although previous studies have demonstrated that under aerobic conditions, the growth of aerobic bacteria or fungi reduces the level of oxygen and thereby facilitates the growth of strictly anaerobic bacteria in both the planktonic and biofilm modes of growth [43,44,45,46,47,48,49], most of these studies were carried out using oral or gut microbes. Bernard et al. [44] showed that three different strains of *Candida albicans* increased biofilm formation by *C. acnes* strains ATCC 6919 (acne isolate) and CE1 (shoulder isolate) by 1-log-unit over 24 h when biofilms were cultured in brain heart infusion broth on polystyrene disks under aerobic conditions. *C. albicans* did not increase *C. acnes* biofilm formation under anaerobic conditions. It is difficult to compare these results to those of the present study because Bernard et al. [44] found that *C. acnes* strain ATCC 6919 alone formed equally robust biofilms under both anaerobic and aerobic conditions. Our findings are the first to demonstrate the aerobic growth of *C. acnes* strains that are unable to grow alone in air, and the first to show that an anaerobic bacterium can outcompete an aerobic bacterium under aerobic conditions.

One possible explanation for the phenotype observed in the present study is that rapidly growing staphylococcal biofilms create anaerobic niches where *C. acnes* is protected from the bacteriostatic effects of oxygen, thereby enabling *C. acnes* to grow. This model is supported by the fact that *S. epidermidis* does not enable the aerobic growth of *C. acnes* in tubes incubated under dynamic conditions (Figure 3a) or in colony biofilms that are exposed to oxygen from both above and below (Figure 5b), and that *aerobic C. acnes*/*S. epidermidis* dual-species biofilms exhibit a bilayer structure, with *C. acnes* evidently growing only at the base of the biofilm (Figure 7). This model is consistent with the observations of Fox et al. [47], who showed that *C. albicans* biofilms created locally hypoxic microenvironments that enabled the growth of anaerobic *Clostridium perfringens* and *Bacillus fragilis* in 6-well, polystyrene microtiter plates under aerobic conditions. Similarly, *C. albicans* biofilms were shown to protect anaerobic *Porphyromonas gingivalis* from unfavorable oxic environments when cultured on glass slides and in 96-well, polystyrene microtiter plates [43], and *Enterococcus faecalis* appeared to shield *P. gingivalis* and support its continued growth in polystyrene microtiter plates under aerobic conditions [49].

Previous studies have shown that poly-*N*-acetylglucosamine exopolysaccharide (PNAG) and double-stranded DNA (eDNA) function as adhesive components in both *S. epidermidis* and *C. acnes* biofilms in vitro [32,33,34]. Although DNase I was shown to detach pre-formed *C. acnes* mono-species biofilms [32,36,39], DNase I did not detach *S. epidermidis* strain 5/*C. acnes* strain HL086PA1 dual-species biofilms (Figure 1c). Instead, the PNAG-degrading enzyme dispersin B efficiently detached *S. epidermidis* strain 5/*C. acnes* strain HL086PA1 dual-species biofilms (Figure 1c), suggesting that PNAG is the major intercellular adhesion on these biofilms. Although *S. epidermidis* strain 5 has been shown to produce PNAG [28], it is not known whether *C. acnes* strain HL086PA1 or other strains used in our study produce PNAG. Therefore, more experiments are needed to determine whether *S. epidermidis* or *C. acnes* PNAG, or both, contribute to biofilm cohesion in *Staphylococcus*/*Cutibacterium* dual-species biofilms.

## 5. Conclusions

Our major finding was that staphylococci enabled *C. acnes* to form luxuriant biofilms under aerobic conditions. This phenotype was exhibited by multiple staphylococcal and *Cutibacterium* species and strains in glass tubes, polypropylene tubes, and polystyrene microtiter plate wells. Confocal fluorescence microscopy revealed that staphylococci may contain anaerobic niches at the base of the biofilm where *C. acnes* can grow. Aerobic *Cutibacterium*/*Staphylococcus* dual-species biofilms provide a novel method for culturing anaerobic *Cutibacterium* spp. in vitro. This dual-species biofilm model may also replicate the symbiotic relationship between *Cutibacterium* and *Staphylococcus* on human skin. It may also offer insight into how *C. acnes* colonizes oxic and moderately hypoxic sites on the skin.

## Figures and Tables

**Table 1:** Bacterial strains.

Species	Strain	Characteristics *	Reference
*C. acnes*	2	Moderate acne, SLST A1, Erm^r^	[25]
*C. acnes*	7	Moderate acne, SLST C1, Erm^r^	[25]
*C. acnes*	14	Mild acne, SLST D1, Erm^r^	[25]
*C. acnes*	HL086PA1	Severe acne, SLST E4, Erm^r^	[26]
*C. avidum*	42	Acne patient, Erm^r^	[25]
*S. aureus*	JE2	Necrotizing fasciitis, MRSA, USA300 clone, Erm^s^	[27]
*S. capitis*	JK9	Healthy subject, Erm^s^	Laboratory strain
*S. capitis*	JK10	Healthy subject, Erm^s^	Laboratory strain
*S. epidermidis*	5	Implant infection, Erm^s^	[28]
*S. epidermidis*	1457	Implant infection, Erm^s^	[29]
*S. epidermidis*	1457/pCM29-GFP	Strain 1457 expressing GFP, Erm^s^	[30]
*S. epidermidis*	JK4	Healthy subject, Erm^s^	Laboratory strain
*S. epidermidis*	JK6	Healthy subject, Erm^s^	Laboratory strain
*S. epidermidis*	JK7	Healthy subject, Erm^s^	Laboratory strain
*S. hominis*	JK5	Healthy subject, Erm^s^	Laboratory strain

* SLST, single-locus sequence type [31]; Erm^r^, erythromycin-resistant; Erm^s^, erythromycin-sensitive; GFP, green fluorescent protein.

## Data Availability

The authors confirm that the data supporting the findings of this study are available within the article. No new datasets were created during the course of the study.
